# Leveraging Smart Telemedicine Technology to Enhance Nursing Care Satisfaction and Revolutionize COVID-19 Care: Prospective Cohort Study

**DOI:** 10.2196/53456

**Published:** 2025-01-21

**Authors:** You-Lung Chang, Chi-Ying Lin, Jiun Hsu, Sui-Ling Liao, Chun-Ti Yu, Hung-Chueh Peng, Chung-Yu Chen, Matthew Huei-Ming Ma, Juey-Jen Hwang

**Affiliations:** 1 Department of Internal Medicine, National Taiwan University Hospital Yunlin Branch, Douliou, Yunlin County, Taiwan; 2 Medical Information Technology Office, National Taiwan University Hospital Yunlin Branch, Yunlin County, Taiwan; 3 Department of Nursing, National Taiwan University Hospital Yunlin Branch, Yunlin County, Taiwan; 4 College of Medicine, National Taiwan University, Taipei, Taiwan; 5 Department of Cardiovascular Medicine, Fu Jen Catholic University Hospital, New Taipei, Taiwan

**Keywords:** COVID-19, telemedicine, smart home care, intelligent medical care, nursing care, medical care, remote monitoring, vital signs, quarantine, home-care, hospital-care, screening, treatment, mHealth, mobile health, digital health, health education, remote equipment, smartphone, video consultation, remote care, caregiver, patient, quality of care, medical staff

## Abstract

**Background:**

Telemedicine has been utilized in the care of patients with COVID-19, allowing real-time remote monitoring of vital signs. This technology reduces the risk of transmission while providing high-quality care to both self-quarantined patients with mild symptoms and critically ill patients in hospitals.

**Objective:**

This study aims to investigate the application of telemedicine technology in the care of patients with COVID-19, specifically focusing on usability, effectiveness, and patient outcomes in both home isolation and hospital ward settings.

**Methods:**

The study was conducted between January 2022 and December 2022. More than 800 cases were monitored using the QOCA remote home care system, a telemedicine platform that enables remote monitoring of physiological data—including heart rate, blood pressure, temperature, and oxygen levels—through Internet of Things devices and a 4G-connected tablet. Of these, 27 patients participated in thie study: the QOCA remote home care system was deployed 36 times in the isolation ward and 21 times to those in home isolation. The QOCA remote care system monitored isolated cases through remote care packages and a 4G tablet. Case managers and physicians provided telemedicine appointments and medications. Innovative methods were developed to enhance usage, including online health education, remote care equipment instructions via QR core links, and video consultations for patients without smartphones.

**Results:**

A clinical nurse satisfaction survey revealed that most respondents found the content of the remote care package comprehensive and the interface easy to learn. They expressed a desire to continue using the system. The majority also agreed that using the remote care system and package would reduce their workload and that patients and caregivers could easily learn to use the package. While some respondents expressed concerns about network and Bluetooth connectivity, the majority (24/27, 89%) agreed to include the remote device as part of their routine equipment, with an average score of 84.8 points.

**Conclusions:**

The integration of telemedicine technology improves the quality of care while reducing the workload and exposure of health care workers to viruses.

## Introduction

COVID-19, a global pandemic, has posed significant challenges to health care systems worldwide [1,2]. While Taiwan has achieved success in epidemic prevention, the reopening of borders raises concerns about potential gaps in prevention measures and strains on health care resources. Addressing these challenges, this study identifies critical needs in the context of COVID-19 care.

The need for home quarantine and home medical care is vital. National Taiwan University Hospital Yunlin Branch has pioneered a telecommunication-based remote consultation model [3], providing video consultations for individuals under home quarantine. This initiative has not only met their basic health care needs but also minimized the risk of infection by reducing hospital visits. However, with the majority of cases being mild or asymptomatic, and a higher proportion of symptomatic cases among middle-aged and older individuals [4], it is crucial to develop a comprehensive telemedicine model for continuous medical care during home quarantine or isolation and for patients with mild COVID-19 undergoing home treatment [5-7].

During the COVID-19 pandemic, home monitoring of patients has gained popularity. There are two main research designs: (1) assessing patients before hospital admission to reduce virus exposure and identify deterioration and (2) postdischarge monitoring to provide continued care [8]. Monitoring methods include online platforms, paper-based operations with telephone interviews, or wearable devices [9-11]. Paper-based operations with telephone interviews are more inclusive due to limited digital literacy among the target population [12,13]. Most studies focus on vital signs rather than psychological issues. Wearing a pulse oximeter is important for monitoring, but it may cause anxiety and has limitations for certain patients [14-16]. Home monitoring during COVID-19 has benefits and can reduce health care costs [17]. Improving accessibility and convenience is key for future adoption.

Due to the pandemic, health care providers are unable to provide patients with the usual standard of care during hospitalization, leading to various challenges and impacts. Health care workers face psychological stress and staffing shortages, while patients experience isolation and mental health issues. Family members are unable to visit their loved ones in critical condition [18,19]. Technology can offer some assistance in addressing these issues, such as self-monitoring of blood oxygen levels and the integration of vital sign monitoring [20]. Collaboration between technology companies and health care providers is crucial, especially as the pandemic becomes normalized in the community. The development of information-assisted solutions is urgent in order to improve health care delivery [21].

There is a need for low-contact health care for hospitalized, isolated patients. To mitigate the risk of infection among patients with confirmed COVID-19 infection or those at high risk of being infected in dedicated isolation wards, the integration of smart ward solutions and telemedicine is proposed. Real-time remote physiological monitoring can minimize exposure risks and reduce the workload of health care staff, optimize management processes, and preserve valuable isolation rooms and personal protective equipment. Furthermore, obtaining real-time patient information enables early intervention and improves work efficiency, thereby enhancing the safety of patients with confirmed COVID-19 infection.

The study was conducted as a collaboration between National Taiwan University Hospital Yunlin Branch and Quanta Computer, utilizing their expertise in big data analysis and telecommunication telemedicine technology. The project focuses on the COVID-19 response hospitals, with the National Taiwan University Hospital Yunlin Branch serving as the testing ground. Specifically, it aims to provide assistance to patients with confirmed COVID-19 infection or those at high risk of being infected in the dedicated isolation ward, using advanced telemedicine equipment for clinical care. By implementing real-time remote physiological monitoring, the project aims to reduce the exposure risk and workload of health care personnel, simplify management processes, and optimize the utilization of valuable isolation rooms and personal protective equipment. Furthermore, by promptly assessing patient conditions and intervening as needed, the project seeks to enhance work efficiency and ensure the safety of patients with confirmed COVID-19 infection.

## Methods

### Ethical Considerations

The Institutional Review Board (IRB) of National Taiwan University Hospital approved this study (202009106RIPA). Questionnaires were collected after obtaining informed consent.

All participant data were anonymized or deidentified to ensure privacy. Participants voluntarily provided informed consent, and no personal identifiers were included in the analysis or dissemination. Additionally, anonymous surveys were used to ensure there were no risks of information leakage.

Participants in the study were not compensated monetarily or otherwise. Their involvement was voluntary, and all necessary support for participation, such as the provision of remote care packages and telemedicine tools, was offered free of charge. This ensured equitable participation without financial coercion.

### Study Design, Data Sources, and Population

The study was conducted at the National Taiwan University Hospital Yunlin Branch. The inclusion criteria comprised individuals diagnosed with COVID-19, while exclusion criteria included moderate to severe COVID-19 cases, patients unwilling to participate in telemedicine monitoring, and individuals deemed unsuitable for telemedicine evaluation by physicians. Participants were required to be 20 years of age or older and provide informed consent approved by the IRB. The study enrollment period spanned from January to December 2022.

### Home Quarantine and Home Medical Care

This study included individuals who have tested positive for COVID-19 and were classified as mild cases or have been determined by the Center for Disease Control, Taiwan, to not require immediate hospitalization due to severe conditions. These individuals can undergo self-isolation monitoring and were either required to quarantine or be monitored at home or quarantine hotels.

**Figure 1.**
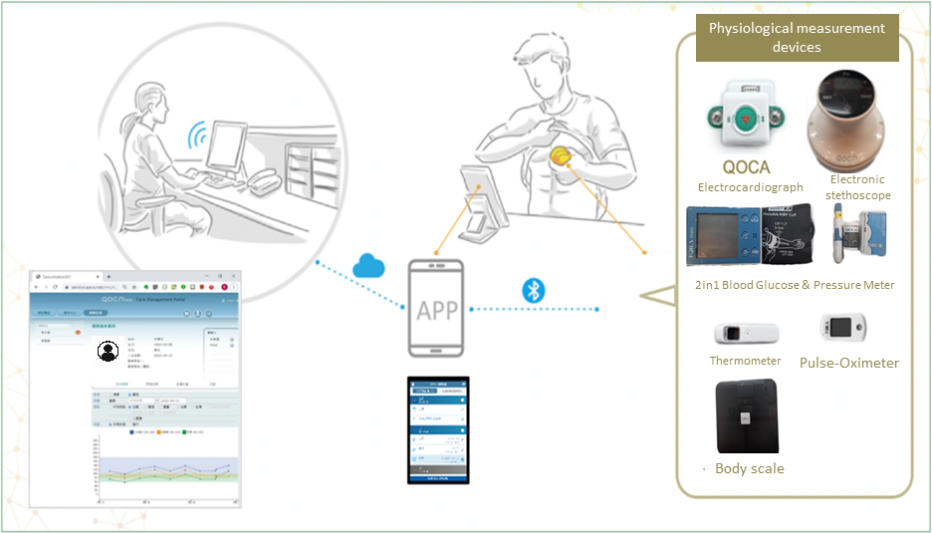
The QOCA home system by Quanta Computer is central to the remote health care platform. Utilizing a 4G tablet, it enables remote communication, physiological data transmission, and virtual exams. Monitoring occurs at homes or quarantine locations, allowing audiovisual contact and basic assessment. IoT devices measure and send vital signs, while an electronic stethoscope captures and uploads lung and heart sounds. An interactive app records visual and clinical data for health care providers. An online questionnaire tracks COVID-19 symptoms, comorbidity control, and psychological well-being and includes medication assessment and personalized care planning. IoT: Internet of Things.

The QOCA home system, developed by Quanta Computer, is the core of the remote health care platform. It utilizes a 4G-connected tablet computer for patients, offering features like remote video communication, physiological signal measurement transmission, and remote physical examination (Figure 1). Home monitoring occurs at the patient’s residence or quarantine hotel, providing audiovisual communication and simple consciousness assessment. Vital signs, such as heart rate, blood pressure, temperature, and oxygen level, are measured and transmitted using Internet of Things (IoT) devices. A remote physical examination system, including electronic stethoscope devices, captures and uploads important findings like lung and heart sounds. An interactive care app records visual aspects and clinical conditions, which can be uploaded for health care providers. An online questionnaire system tracks COVID-19 symptoms, comorbidity control, and psychological well-being. Medication assessment and personalized care planning are provided. The hospital-side system manages home isolation quarantine and home-based COVID-19 care. Case managers track patient conditions through a dashboard, maintain daily health logs, and determine if hospital treatment is needed. Information and communication technology–based remote health care ensures continuous medical care and psychological support for individuals in home quarantine or isolation and home-based patients with COVID-19. Remote care packages enable self-isolated individuals to monitor and report symptoms and vital signs, with data transmitted back to the hospital. Dedicated care managers communicate with patients, report to physicians, and provide necessary support in case of discomfort or abnormalities.

### Smart Hospital Ward

Patients with COVID-19 admitted to negative pressure isolation wards or intensive care units at National Taiwan University Hospital Yunlin Branch were monitored using the QOCA home system developed by Quanta Computer. Tablet computers, IoT physiological monitoring devices, and video cameras were installed in patient rooms for remote monitoring. Patient physiological data, including heart rate, respiration, body temperature, blood pressure, and blood oxygen levels, were automatically recorded and uploaded to the cloud. The care team accessed these data without entering the rooms. The smart ward included an electronic stethoscope device for recording heart and lung sounds. A central monitoring module and a patient dynamic dashboard were established at the nursing station, providing alerts for abnormal conditions. Patient laboratory reports and images were integrated, enabling health care providers to interact with patients through the video system. The study aimed to utilize information and communication technology, including 4G-connected tablets and IoT monitoring platforms, to facilitate remote health care for patients with COVID-19 in isolation units. This system enabled continuous medical care, psychological support, and remote consultations with physicians; nursing care; psychological counseling; and rehabilitation therapy. It allowed for monitoring of vital signs and COVID-19 symptoms, promoting patient well-being without direct contact.

### Satisfaction Survey for the QOCA Smart Ward Remote Care System: Clinical Nursing Staff and General Population

This questionnaire aimed to understand the relevant settings of the remote care system and its clinical application in dedicated wards for COVID-19. The information gathered will be used for future improvements and optimization. This survey was anonymous, and there were no concerns about personal information leakage. The questionnaire included aspects of communication quality, user-friendliness, overall satisfaction, and suggestions. Each option consisted of strongly agree, agree, neutral, disagree, and strongly disagree (Multimedia Appendix 1).

## Results

### Overview

Before officially enrolling patients in the monitoring care package, thorough explanations and assistance were provided to each individual in the isolation ward to ensure their understanding and obtain their consent to participate in the research study. Following this, every isolated patient was equipped with a set of remote care packages. This became standard practice in the ward, significantly reducing the frequency of health care personnel entering patient rooms to don and remove isolation garments. Patients were empowered to self-measure their vital signs whenever they felt unwell, providing immediate access to physiological data that could alert health care professionals to any discomfort or changes in their condition. This approach not only alleviated patients’ psychological anxiety about their disease progression but also allowed health care staff to spend less time in isolation garments while maintaining a real-time understanding of the patients’ current status, ultimately improving the quality of clinical care.

### Enhancing Hardware Infrastructure for Remote Care System in Isolation Ward

To enhance the hardware aspect of the remote care system implemented in the isolation ward, the original 7-bed negative pressure ward was expanded to accommodate the installation of 32 sets of care packages, with one set allocated to each bed. Additionally, the nursing treatment cart and the nursing station computer were equipped with the QOCA remote care system platform and individual nursing staff accounts. This setup empowered the nursing personnel to monitor the patients’ vital sign data in real time, which were automatically uploaded by the system after patient measurements. Continuous monitoring of vital signs was made possible throughout the patients’ entire hospitalization and isolation period. Moreover, the QOCA system facilitated real-time video communication when necessary, enabling consultations with psychiatric physicians to address any illness-related anxieties that the patients may have. By extending the focus of care beyond the patients’ physiological needs, the hospitalization and treatment process also addressed their psychological well-being.

### Remote Home Care Package for Individual Who Are COVID-19 Positive Under Self-Isolation

For individuals who have tested positive for COVID-19 and can self-isolate at home without requiring hospitalization, a comprehensive remote home care package was provided. This package enabled individuals to independently monitor their health, even without monitoring devices at home. It included daily self-observation of symptoms and measurement of vital signs, with the numerical data automatically transmitted to the hospital through a tablet computer connected to 4G.

To ensure prompt attention to any discomfort or adverse conditions, a dedicated care manager was assigned to each patient. The care manager promptly reported any concerns to the responsible physician and maintained regular contact to inquire about the patient’s condition throughout the home isolation period. If individuals experience symptoms, they could also schedule telemedicine appointments to closely monitor changes in their condition.

In cases where symptom management was necessary, the responsible physician could prescribe appropriate medications to help alleviate symptoms. This comprehensive remote home care package aimed to provide effective care and support to individuals with COVID-19, ensuring their well-being while minimizing the risk of further transmission.

### Advancing Care Through QOCA Telecare System for COVID-19 Monitoring and Isolation

The number of cases monitored using the QOCA remote home care system exceeded 800. Among them, 29 individuals voluntarily participated in this research study and provided their consent by signing the IRB consent form. The QOCA remote home care system was deployed 36 times in the isolation ward and 21 times to those in home isolation, as indicated in Table 1. In the ward, patients primarily monitor themselves or receive assistance from family members for monitoring, while a small number of individuals without family support or hired caregivers receive help from nursing staff for measurements.

The participants in this study represented a diverse range of age groups, with the majority being aged 41-60 years (13/29, 45%), followed by the 61-80 years age group (9/29, 31%), and the fewest participants in the 21-40 years (3/29, 10%) and 80+ years age groups (4/29, 14%). The sample was predominantly male, with 27 (93%) male participants and only 2 (7%) female participants. Regarding the need for assistance, 11 (38%) participants required help during the study, while 18 (62%) managed independently. The participants’ education levels varied, with most having elementary or junior high education (20/29, 69%), while a smaller number had completed high school or higher education (9/29, 31%). In terms of technology usage, 21 (72%) participants used digital devices daily, while 8 (28%) reported no prior experience. Health engagement levels were mixed, with the majority (20/29, 69%) not actively seeking information about their conditions, while some participants regularly consumed health-related content. Additionally, 17 (59%) participants had prior experience with health monitoring devices, further illustrating the range of familiarity with telecare technology among the group.

In contrast to traditional inpatient monitoring methods, the ward implemented a specialized remote home care system designed specifically for the isolation care required during the COVID-19 pandemic. This system enables patients to independently measure their vital signs and automatically transmit the data to the nursing staff. To ensure effective utilization of the remote home care system by patients and their accompanying family members, innovative care methods have been developed, including the following:

1. Online health education materials: With the widespread use of smartphones due to technological advancements, disease-specific health education materials are provided through QR codes during hospitalization. This allows patients to access relevant information whenever needed.
2. Guidelines for operating remote care devices: Some individuals may be unfamiliar with monitoring devices. Even after receiving instructions from health care professionals, patients may require repeated practice. To facilitate practice sessions without overwhelming the nursing staff, instructional videos demonstrating device operation can be accessed through QR codes. Patients can practice as many times as necessary to ensure accurate measurements.
3. Tablet-based video consultations for remote care: Patients without smartphones can still access important information through tablet-based video consultations offered by the remote care system.

The ward continues to fully utilize the remote home care system for clinical monitoring, and the number of users continues to grow. Health care professionals not only provide personalized care to patients but also make adjustments to the care process based on the implementation of the remote home care system. This approach reduces the risk of viral exposure and eases the workload for health care staff, streamlines management procedures, and ultimately enhances the quality of care provided.

**Table 1.** Results of case enrollment.

	Value
**QOCA remote home care system, n**	
Isolation ward	36
Home isolation	21
Total	57
Goal	50
**Total utilization (cases), n**	>800
**Execution rate**	100%

### Clinical Nursing Staff Satisfaction Survey

This survey aimed to assess the satisfaction and experiences of the clinical nursing staff in using the telecare system in the dedicated COVID-19 isolation wards. The questionnaire was divided into 3 main categories: *communication quality*, *user-friendliness*, and *overall experience*. The objective was to identify and analyze the challenges and difficulties faced by users in relation to equipment, signals, and interface operations.

A total of 27 questionnaires were collected exclusively from nursing staff working in the dedicated COVID-19 wards, specifically Ward 7A and Ward 7B. Regarding the transmission signals of the equipment, 44% (n=12) of the respondents believed that the internet connection of the telecare system was stable, while 37% (n=10) found the Bluetooth connection between the tablet and the devices in the package to be stable. When using the telecare system for video calls, 56% (n=15) stated that the image quality was clear, and 56% (n=15) stated that the sound quality was clear (Table 2).

In terms of usability, following training on the telecare system’s interface, 89% (n=24) of the respondents found it easy to learn, and an equal percentage (n=24, 89%) expressed their willingness to continue using the telecare system to assist in clinical care. Furthermore, 67% (n=18) believed that patients or caregivers could quickly grasp the usage of the telecare package. Moreover, 81% (n=22) of the respondents indicated that the telecare package contained all the necessary components and fulfilled the requirements for clinical monitoring. Additionally, 89% (n=24) agreed that the use of the telecare system and package could help alleviate the burden of clinical care (Table 3).

Overall, 89% (n=24) of the respondents agreed to incorporate the telecare device as a regular piece of equipment in isolation wards, with an average satisfaction score of 84.8. The majority (24/27, 89%) of the responses highlighted the short monitoring time of the device and the instability of signal transmission. They expressed a desire for improved stability in wireless internet and Bluetooth signals, as it would lead to a smoother user experience (Table 4).

**Table 2.** Communication quality survey responses regarding the telemedicine system.

Question	Score^a^ (n=27), n (%)				
	1	2	3	4	5
I think the network connection of this telecare system is stable	1 (4)	11 (41)	12 (44)	3 (11)	0 (0)
The Bluetooth connection between the tablet in the care bag and the instrument is stable	2 (7)	8 (30)	12 (44)	5 (19)	0 (0)
When using the nursing system, the image is very clear.	3 (11)	12 (44)	6 (22)	5 (19)	1 (4)
When using the nursing system, the sound is very clear.	2 (7)	13 (48)	8 (30)	3 (11)	1 (4)

a Score: 1=strongly agree, 2=agree, 3=neutral, 4=disagree, and 5=strongly disagree.

**Table 3.** User-friendliness survey responses regarding the telemedicine system.

Question	Score^a^ (n=27), n (%)				
	1	2	3	4	5
The interface of this telecare system is easy for me to use after being taught.	15 (56)	9 (33)	1 (4)	1 (4)	1 (4)
I find the equipment in this telecare package difficult to use.	1 (4)	3 (11)	1 (4)	6 (22)	16 (59)
I think most patients or caregivers can quickly learn to use the telecare package.	7 (26)	11 (41)	4 (15)	3 (11)	2 (7)
I think the contents of this telecare package are complete and meet the needs of clinical monitoring	12 (44)	10 (37)	3 (11)	1 (4)	1 (4)

a Score: 1=strongly agree, 2=agree, 3=neutral, 4=disagree, and 5=strongly disagree.

**Table 4.** Overall satisfaction with the QOCA remote care system in smart wards.

Question and score	Value (n=27), n (%)
**I think the use of telecare systems and care packages can help reduce the load of clinical care.^a^**	
1	15 (56)
2	9 (33)
3	2 (7)
4	0 (0)
5	1 (4)
**I would like to continue to use this telecare system to assist clinical care.^a^**	
1	15 (56)
2	9 (33)
3	2 (7)
4	0 (0)
5	1 (4)
**I think the remote care system and care package can become one of the routine equipment used by the unit.^a^**	
1	15 (56)
2	6 (22)
3	4 (14)
4	1 (4)
5	1 (4)
**If a full score is 100, how much would you like to rate the telecare system and care package?**	
60	1 (4)
70	2 (7)
75	1 (4)
80	6 (22)
85	3 (11)
90	11 (41)
95	2 (7)
99	1 (4)

a Score: 1=strongly agree, 2=agree, 3=neutral, 4=disagree, and 5=strongly disagree.

## Discussion

### Principal Findings

The enhancement of the hardware infrastructure for the remote care system in the isolation ward has significantly improved the quality of care and patient outcomes [22,23]. By expanding to accommodate more care packages, each patient receives continuous monitoring and support. The integration of the QOCA remote care system into nursing carts and computers allows real-time access to vital sign data, enabling prompt interventions and ensuring patient well-being. The system also facilitates video consultations with psychiatric physicians, addressing patients’ anxieties and holistic needs. This approach benefits both patients and nursing staff, who can efficiently monitor multiple patients, respond promptly to changes, and access patient data remotely, thus saving time and reducing errors.

The implementation of a remote home care package for individuals who are COVID-19 positive in self-isolation offers effective care and support while reducing transmission risk [24]. Patients can independently monitor their health by observing symptoms and measuring vital signs, actively participating in their care. A tablet connected to 4G enables real-time transmission of data to health care providers, facilitating informed decisions. Dedicated care managers provide personalized support, regularly checking in on patients and promptly reporting concerns. Telemedicine appointments allow close monitoring, adjustment of treatment plans, and direct access to medical professionals, thus reducing anxiety. Remote prescription of medications ensures symptom management without visiting a health care facility. However, challenges include subjective self-reporting and disparities in internet access and technological skills [23,25]. Education and efforts to address disparities are essential for equitable care.

The implementation of the QOCA remote home care system for COVID-19 monitoring and isolation has been successful in advancing care and improving patient outcomes. With over 800 cases monitored, the system allows patients to independently measure vital signs and transmit data to nursing staff, reducing the need for frequent in-person monitoring. Innovative methods, such as online health education materials and instructional videos, ensure optimal utilization of the system. Tablet-based video consultations cater to patients without smartphones. By fully utilizing the remote home care system, the ward provides personalized care, streamlines management procedures, reduces viral exposure, and eases the workload for staff, ultimately enhancing care quality [26]. Ongoing monitoring and evaluation are crucial for system refinement, and its success in COVID-19 care opens doors for its application in other health care settings, showcasing the transformative potential of technological advancements in patient care.

During the COVID-19 pandemic, nursing professionals have made substantial achievements in delivering adequate patient care by utilizing telehealth [8,12,24]. The results of the clinical nursing staff satisfaction survey regarding the telecare system in COVID-19 isolation wards provide valuable insights into their experiences and challenges [27]. The majority of respondents found the telecare package to be usable and to have met the requirements for clinical monitoring. After training, the respondents stated that the interface was easy to learn, and they expressed their willingness to continue using the system. A substantial percentage believed that patients and caregivers could quickly grasp its usage, and that the use of the telecare system helped alleviate the burden of clinical care [12]. Regarding transmission signals, opinions were mixed. While a portion of respondents found the internet connection stable, others highlighted issues with Bluetooth connectivity. In terms of video calls, a majority found the image and sound quality to be clear. Overall, the survey indicates high satisfaction with the telecare system, with a majority of respondents expressing their support for incorporating it as a regular equipment in isolation wards. The average satisfaction score was positive [13]. However, feedback emphasized the need for improvements in signal stability, particularly in wireless internet and Bluetooth connections [24]. The short monitoring time of the device was also a concern.

The difficulties and obstacles in telemedicine for COVID-19 care include insufficient hardware environment and the short monitoring time of equipment [28]. The negative pressure isolation environment in the ward, with multiple walls and controlled doors, leads to weak signals. Installing signal amplifiers, as suggested by the hospital’s IT department, can improve signal stability. The monitoring time of the equipment is too short, but through discussions with engineers and the manufacturer, it is believed that adjusting the monitoring time according to individual patient needs can align it with clinical requirements, enabling more accurate monitoring of values. Addressing these challenges will enhance the effectiveness and reliability of telemedicine in COVID-19 care.

One potential limitation of this study is the relatively small sample size, which may affect the generalizability of the findings. With only 36 deployments in the isolation ward and 21 deployments in home isolation, the results may not fully represent the broader population of patients with COVID-19 or other health care contexts. Additionally, the voluntary nature of participation could introduce selection bias, as those who agreed to participate may differ from those who declined in terms of health status or access to technology. Another limitation is that the study was conducted within a single hospital, potentially limiting the applicability of the results to other settings with different resources or patient demographics. Lastly, the short-term focus of the study may not capture the long-term effectiveness or sustainability of telemedicine interventions, warranting further research in this area.

### Conclusion

The implementation of the QOCA telemedicine system demonstrated significant benefits in managing patients with COVID-19 who have mild symptoms, both in isolation wards and home quarantine settings. By enabling continuous remote monitoring of vital signs and offering real-time communication between patients and health care providers, the system not only improved the quality of care but also reduced the workload and exposure risk for medical staff.

However, the study had some limitations, including a relatively small sample size and the potential for selection bias, which may affect the generalizability of the findings. Additionally, the short-term nature of the study may not fully capture the long-term impacts of telemedicine in patient care.

Despite these limitations, our findings suggest that telemedicine systems like QOCA can play a crucial role in pandemic response and future health care delivery. Further research with larger, more diverse populations and extended follow-up periods is recommended to fully explore the potential of telemedicine in improving patient outcomes and health care efficiency.

## Abbreviations

IoT: Internet of Things
IRB: institutional review board
